# From Insulator to Superconductor: A Series of Pressure-Driven Transitions in Quasi-One-Dimensional TiS_3_ Nanoribbons

**DOI:** 10.1021/acs.nanolett.4c00824

**Published:** 2024-04-29

**Authors:** Mahmoud Abdel-Hafiez, Li Fen Shi, Jinguang Cheng, Irina G. Gorlova, Sergey G. Zybtsev, Vadim Ya. Pokrovskii, Lingyi Ao, Junwei Huang, Hongtao Yuan, Alexsandr N. Titov, Olle Eriksson, Chin Shen Ong

**Affiliations:** § Center for Advanced Materials Research, Research Institute of Sciences and Engineering, University of Sharjah, Sharjah, United Arab Emirates; € Department of Applied Physics and Astronomy, University of Sharjah, P.O. Box 27272 Sharjah, United Arab Emirates; ± Department of Physics and Astronomy, Uppsala University, Box 516, SE-751 20 Uppsala, Sweden; $ Beijing National Laboratory for Condensed Matter Physics and Institute of Physics, Chinese Academy of Sciences, Beijing 100190, China; # School of Physical Sciences, University of Chinese Academy of Sciences, Beijing 100190, China; ⊥ Kotelnikov Institute of Radioengineering and Electronics of RAS, 125009 Moscow, Russia; ‡ National Laboratory of Solid State Microstructures, College of Engineering and Applied Sciences and Jiangsu Key Laboratory of Artificial Functional Materials, Nanjing University, Nanjing 210000, China; ∇ M.N. Miheev Institute of Metal Physics of Ural Branch of Russian Academy of Sciences, 620990 Yekaterinburg, Russia; ▼ WISE-Wallenberg Initiative Materials Science, Uppsala University, SE-751 20 Uppsala, Sweden

**Keywords:** Superconductivity, quasi-one-dimensional materials, transition metal trichalcogenides, pressure, crystal structure, phase transitions

## Abstract

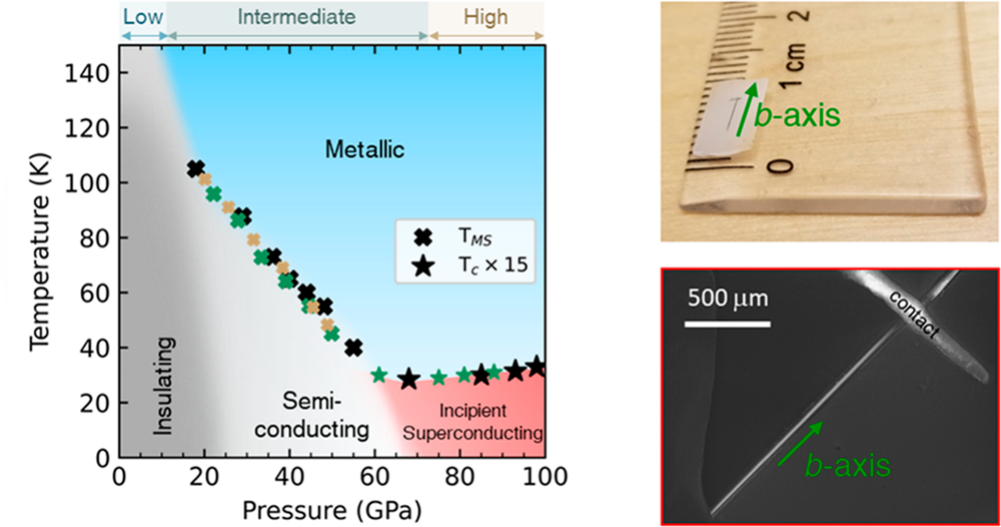

Transition metal trichalcogenides (TMTCs) offer remarkable opportunities for tuning electronic states through modifications in chemical composition, temperature, and pressure. Despite considerable interest in TMTCs, there remain significant knowledge gaps concerning the evolution of their electronic properties under compression. In this study, we employ experimental and theoretical approaches to comprehensively explore the high-pressure behavior of the electronic properties of TiS_3_, a quasi-one-dimensional (Q1D) semiconductor, across various temperature ranges. Through high-pressure electrical resistance and magnetic measurements at elevated pressures, we uncover a distinctive sequence of phase transitions within TiS_3_, encompassing a transformation from an insulating state at ambient pressure to the emergence of an incipient superconducting state above 70 GPa. Our findings provide compelling evidence that superconductivity at low temperatures of ∼2.9 K is a fundamental characteristic of TiS_3_, shedding new light on the intriguing high-pressure electronic properties of TiS_3_ and underscoring the broader implications of our discoveries for TMTCs in general.

Transition metal chalcogenides are chemical compounds that consist of at least one chalcogen anion and one electropositive element. Unlike their transition metal dichalcogenides cousins, which have higher degrees of in-plane symmetry, TMTCs distinguish themselves with strong in-plane anisotropy of the crystal structure: although the layers can be clearly defined parallel to the *a*–*b* plane (Figure 1A), atoms of the metal form chains, typically along the *b*-direction (Figure 1A,B). Structural anisotropy at the atomic level shows up on the macroscopic level through preferential growth along the *b*-axis (Figure 1B), resulting in distinctive needle-like microstructures known as whiskers or (nano)ribbons, as well as related anisotropic physical properties, which can be observed at room temperature and ambient pressure (see e.g., refs (1−3)). A representative quasi-one-dimensional (Q1D) TMTCs is titanium trisulfide,^1,2,4−15^ TiS_3_.

**Figure 1:**
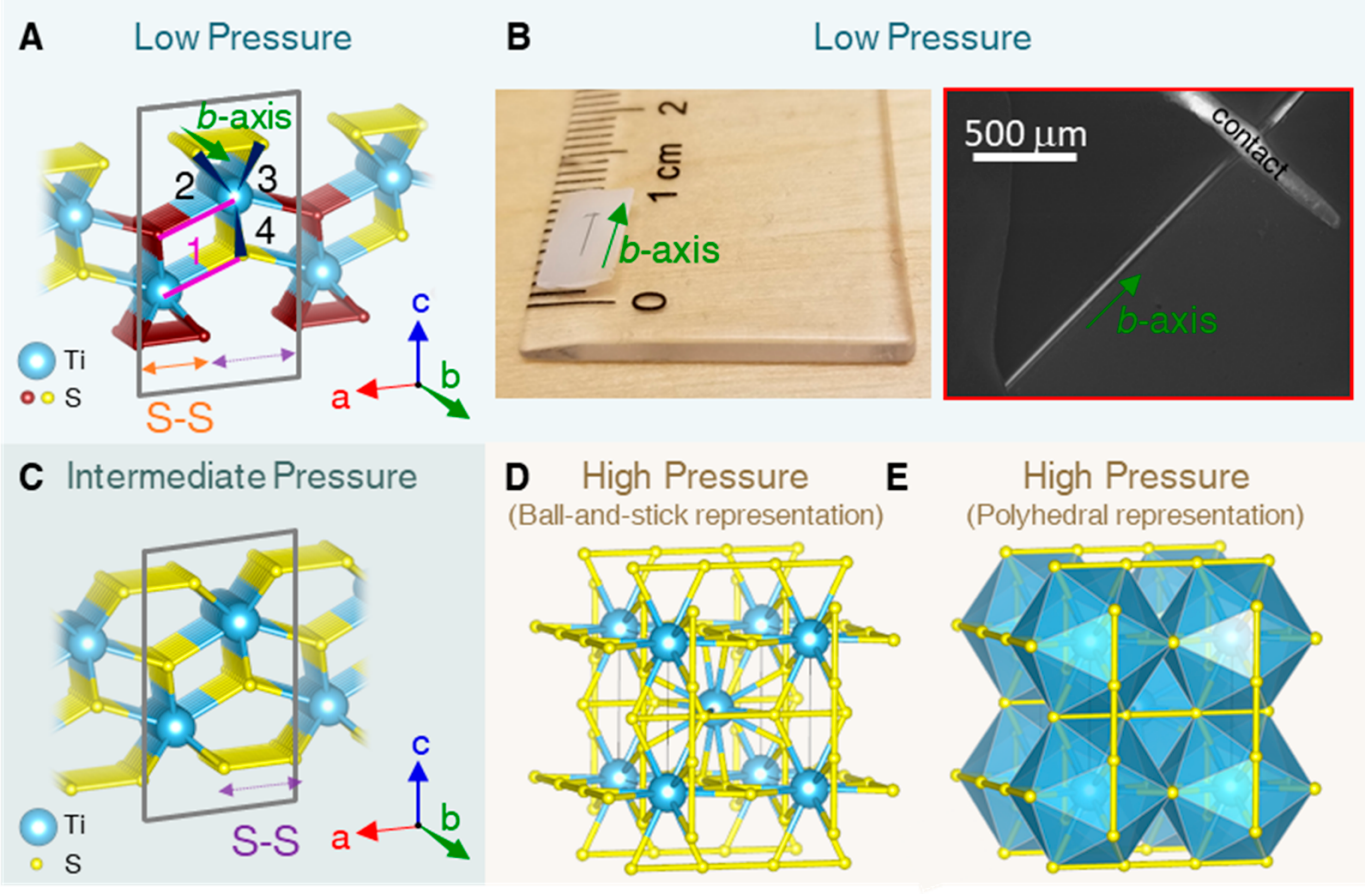
Pressure-induced phases of TiS_3_. (A) Monoclinic crystal lattice of TiS_3_ (space group of *P*2_1_/*m* (type-I)) at low pressure. The gray box outlines the periodic unit cell. Bond 1 (magenta) is 2.67 Å long, while the bonds 2, 3, and 4 (dark blue) are 2.49 Å long on average. To show clearly the embedded 1D chains, we use maroon and yellow S atoms to differentiate the two different (but equivalent) chains within a periodic unit cell. The S–S pair (labeled in orange) connects S atoms attached to the same Ti. (B) *Left*: A photograph of a Q1D TiS_3_ microstructure (seen as a dark line) on a white paper. *Right*: SEM image of the TiS_3_ whisker at low pressure. (C) Monoclinic crystal lattice of TiS_3_, *P*2_1_/*m* (type-II), at intermediate pressure. The gray box outlines the periodic unit cell. The S–S bond (labeled in purple) connects S atoms attached to different Ti. (D) Cubic crystal lattice of the high-pressure phase (space group of *Pm*3*m*) in the ball-and-stick representation. (E) Cubic crystal lattice in the polyhedral representations.

The influence of high-pressure compression on the electronic properties of Q1D materials remains poorly explored. Additionally, it is important to investigate how temperature and pressure affect the vibrational properties of these Q1D structures. At ambient conditions, the crystal structure of TiS_3_ combines strong covalent bonds and weak van-der-Waals-like interactions. The structural unit of TMTC is covalently bonded. We note that the in-plane lattice parameters,^4^*a* = 5.01 Å and *b* = 3.40 Å, reflect the in-plane anisotropy.^2^ Our calculations based on density functional theory (DFT) gave similar results (*a* = 5.03 Å, *b* = 3.42 Å) and also showed that Ti–S bonds with a directional component along the *a*-axis are 2.67 Å long. In Figure 1A, they are outlined in magenta and labeled “1”. These bonds are weaker than the Ti–S bonds shown in dark blue and labeled “2”, “3”, and “4” in Figure 1A. The latter have a directional component along the *b*-axis and an average length of 2.49 Å, consistent with ref (16). From this perspective, the structure of TiS_3_ can be described as consisting of strongly bonded 1D TiS_3_ chains embedded within layers. We note that the S–S bonding (shown in orange in Figure 1A) means that at ambient pressure, TiS_3_ can also be written as [Ti]^4+^[S]^2–^[S_2_]^2–^: every three S atoms accept four electrons from Ti, leaving Ti with no free electrons and making TiS_3_ a semiconductor.^17−19^ The unbuckling of the [S_2_]^2^ pair under pressure (Figure 1C) characterizes a structural phase transition, which is isosymmetric,^14^ i.e., the transition preserves the structural space-group symmetry (monoclinic *P*2_1_/*m*) of the crystal. This phase transition roots in the unique bonding of the S atoms, which are sensitive to subtle variations in interatomic distances.^17,20,21^

The band gap of TiS_3_ at ambient pressure has been widely studied. TiS_3_ has an optical band gap of about 1 eV,^11−13^ approximately the same as in silicon. The temperature dependences of the resistance and Hall concentration of carriers^14,15^ are characterized by an activation energy of 35–40 meV corresponding to the energy of the donor level.^6,7,17,18^ It has been suggested that TiS_3_ exists naturally as an *n*-type semiconductor due to native defects,^7,18^ and the metallic behavior reflects the growth of electronic mobility with *T* reduction. In this scenario, the metal-to-dielectric transition is a just formal convention resulting from the interplay of carrier mobility (e.g., from phonon contributions) and carrier concentration and their dependences on temperature. Alternatively, some authors (e.g., refs (3 and 5)) have argued that near room temperature, TiS_3_ is a degenerate semiconductor, exhibiting characteristics akin to metallicity. In this context, the transition from metal to dielectric is true.

In ref (14), TiS_3_ was studied under pressures of up to 30 GPa (for the XRD structural studies) and 39 GPa (for the transport measurements). Further compression of the microstructures has the potential to reveal new phases such as superconductivity. For materials in quasi-2D and bulk forms, pressure-induced metallization and superconductivity^22−24^ are well-known topics of interest. For 1D microstructures, experimental realization appears to be formidable at the outset. Early studies of other TMTCs^25−31^ under pressure have revealed superconductivity in NbS_3_ (of an uncertain metallic phase^32^), NbSe_3_, TaS_3_, TaSe_3_, and ZrTe_3_. All these compounds have free electrons, at least, above the charge-density wave (CDW) transitions temperatures. Pressure-induced superconductivity has been reported^33^ also for the triclinic phase of NbS_3_, which is semiconducting at ambient conditions. However, its semiconducting state can be attributed to the dimerization of the structure, i.e., a form of CDW, which is suppressed under moderate pressure.^34^ TiS_3_ is intrinsically insulating in the absence of CDW at room temperature and ambient pressure^18−20^ and, thus, is essentially different from these metals. It has never yet been identified as a superconductor under any conditions.

However, as mentioned above, in TMTCs, a slight variation of the interatomic distance between S atoms can switch them between isolated and coupled states.^17,20,21^ In addition, possible structural transitions under pressure can make TiS_3_ more isotropic. In fact, prior to the present work, through computational structural searches, Zhong et al.^35^ have predicted that application of pressure can induce a structural phase transition of TiS_3_ into the metallic state with cubic crystal structure (Figure 1D,E). Hence, one may hypothesize that under hydrostatic pressure, TiS_3_ may possibly transform its electronic and even crystal structure. Further, Yue et al.^36^ found the first TMTC, HfS_3_, that becomes superconducting under pressure of higher than 50.6 GPa, reaching a superconducting transition temperature, *T*_c_, of 8.1 K at 121 GPa, despite being insulating at ambient pressure. With this in mind, we decided to probe TiS_3_ experimentally under extreme hydrostatic pressure, *P*, at different temperatures, tracking its transport properties as a function of *P* and *T*, supported by the calculations based on first-principles DFT and density functional perturbation theory (DFPT) (see Supporting Information Sec. S1 for computational details).

The details of the growth of the TiS_3_ microstructures can be found in Sec. S2 of the Supporting Information. To control the crystal morphology of TiS_3_, we carried out *in situ* angle-resolved Raman measurements at room temperature (Figure 2). The detection and excitation polarizations of the electric field were both directed perpendicular to the *b*-axis. At ambient pressure, the Raman spectra confirmed the results of previous studies.^4,37−39^ The spectra of TiS_3_ under various pressures up to 35.3 GPa are shown in Figure 2A. The positions of the four main Raman peaks and their split-off peaks were identified using Lorentzian fittings (as exemplified in Figure 2B) and plotted in Figure 2C. A few points should be noted. First, the peaks I, II, and III shifted toward higher frequency (blue shift) as pressure increased. The blue shifts under pressure demonstrated pressure-induced enhancements of the interlayer interactions and intralayer coupling between chains, leading to the stiffening of the atomic bonds and strengthening of the chain-like structures. Among these modes, the *A*_g_^rigid^ (I) mode was the most sensitive to the applied pressure. Second, in contrast to peaks I, II, and III, the fourth *A*_g_^S–S^ (IV) mode was notably red-shifted as pressure increased. The softening of this mode can be attributed to the weakening of the bond between the dangling S–S pair along the *a*-axis, prior to the separation of the S atoms. The above two trends continued as pressure increased up to the highest pressure achievable for our *in situ* pressure-dependent Raman measurements, i.e., 35.3 GPa. Third, as pressure increased from 0.0 GPa, the *A*_g_^internal^ (III) mode split at 4.4 GPa, into two peaks, which merged again at 24.1 GPa. Above 25.5 GPa, the *A*_g_^internal^ (II) and *A*_g_^S–S^ (IV) modes split. The newly created daughter peaks followed the blue and red shifts of their parent peaks. The splitting of the modes was due to the breaking of their double degeneracy under pressure, but without change in the bonding for each Ti atom.

**Figure 2:**
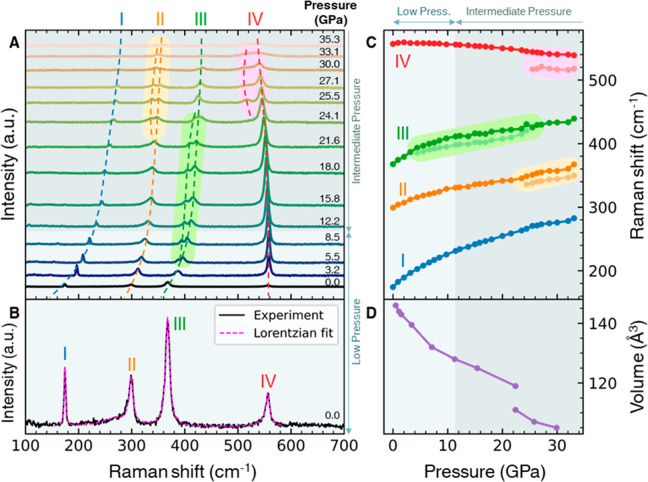
*In situ* angle-resolved Raman spectra at room temperature in the low-to-intermediate pressure regimes. (A) Raman spectra of TiS_3_ at different pressures (0.0 to 35.3 GPa). The four dashed lines trace the peak positions identified through Lorentzian fitting of experimental Raman spectra. Peaks *A*_g_^rigid^ (I), *A*_g_^internal^ (II), *A*_g_^internal^ (III), and *A*_g_^S–S^ (IV) are shown using blue, orange, green and red lines, respectively. The regions where peaks II, III, and IV split are highlighted. (B) Raman spectrum from (A) at ambient pressure. The pink dashed line shows the Lorentzian fits for all the peaks. (C) Frequencies of the modes as a function of pressure. The color for each peak repeats the color of the corresponding dashed line in (A). (D) The unit-cell volume versus pressure found from XRD studies. Arranged from ref (14) with permission. Copyright 2017 American Physical Society.

This unbuckling of the S–S pair characterizes the isosymmetric structural transition from monoclinic *P*2_1_/*m* (type-I) (Figure 1A) to monoclinic *P*2_1_/*m* (type-II) (Figure 1C) structure^13^ as described earlier. Above ∼33 GPa, all prominent Raman peaks were suppressed. In this pressure range, TiS_3_ existed purely in the type-II phase (Figure 2A), which was Raman inactive due to asymmetric vibrations and reduced polarizability of the *P*2_1_/*m* (type-II) structure. The nature of this structural transition was clarified by the high-pressure synchrotron XRD of An et al.:^14^ a big and discontinuous drop in crystal volume was reported at 22.4 GPa, marking the onset of a structural transition from *P*2_1_/*m* (type-I) to the *P*2_1_/*m* (type-II). The curve from ref (14) is reproduced in Figure 2D. Our calculations also showed that at low pressure (Figure 3A, shaded pale blue), the monoclinic *P*2_1_/*m* (type-I) crystal phase of TiS_3_ (Figure 1A) had the lowest enthalpy. As pressure increased, the enthalpy of the low-pressure phase increased faster than the enthalpy of the intermediate-pressure phase such that at pressures above 11.5 GPa (and below 73.2 GPa), the intermediate-pressure phase had the lowest calculated enthalpy instead (Figure 3A, shaded teal green), in agreement with refs (14 and 35). As the applied pressure continued to increase above 40 GPa, the enthalpy of the high-pressure cubic phase decreased relative to the other phases, and for pressure larger than 73.2 GPa (Figure 3A, shaded beige), the high-pressure phase had the lowest enthalpy. This led to a crystal phase transition from the *P*2_1_/*m* (type-II) phase to the cubic phase. The low- (0.0 to 11.5 GPa), intermediate- (11.5 to 73.2 GPa), and high-pressure (73.2 to 98.0 GPa) regimes are defined using this enthalpy plot (Figure 3A) and are, respectively, represented using pale blue, teal green, and beige colors in all figures. The high-pressure phase belongs to the space group *Pm*3*m*. Incidentally, unlike the intermediate-pressure phase, the high-pressure phase satisfies all of the Matthias rules^40^ (see Supporting Information Sec. S3 for elaboration).

**Figure 3:**
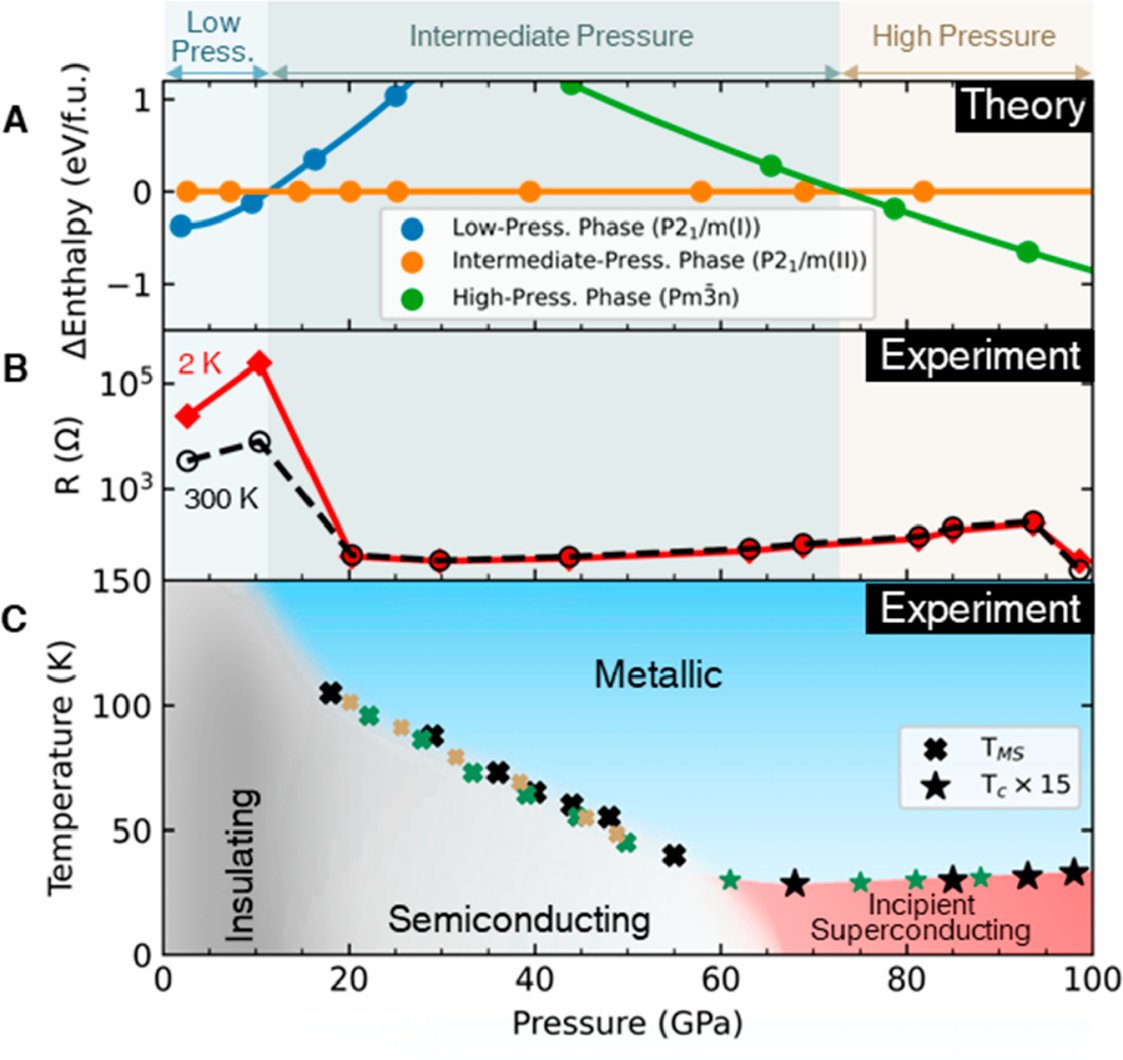
Pressure-induced phase transitions. (A) Calculated enthalpy per formula unit (f.u.) (Ti_2_S_6_) of the three crystal phases, relative to the enthalpy of the intermediate-pressure phase, at different pressures. Pale blue, teal green, and beige plot backgrounds, respectively, denote the low-, intermediate-, and high-pressure regimes. (B) Experimental *R*(*P*) curves of Sample 3 at 2 and 300 K. (C) Temperature–pressure phase diagram based on resistance measurements on three different samples. The metallic, semiconducting, insulating, and superconducting phases are, respectively, represented by blue, light gray, dark gray, and red regions. The metal-to-semiconductor transition (crossover) temperatures, *T*_MS_, are represented by “cross” symbols. The metal-to-superconductor transition temperatures, *T*_c_, amplified by a factor of 15, are represented by “star” symbols. The transition temperatures for Samples 1, 2, and 3 are represented in green, black, and brown, respectively.

The high-pressure resistance of the TiS_3_ microstructures was measured by the standard four-probe method in a nonmagnetic Cu–Be diamond anvil cell. (For details of high-pressure setup and transport measurements, see Supporting Information Sec. S4.) The sequence of changes in electronic orders under increasing pressure, starting from an insulating state and ending with an incipient superconducting state, is effectively depicted by plotting the resistance, *R*, as a function of *P* and *T*, as shown in Figure 3B. Figure 3B shows the resistance-vs-pressure, *R*(*P*), plots of TiS_3_ at 2 and 300 K. These curves support the calculated enthalpy-vs-*P* diagram in Figure 3A. To obtain the full phase diagram, we systematically studied the *R*-vs-*T* behavior, from 1.5 to 300 K in different pressure regimes, from ambient pressure (Figure 4A) up to 94 GPa (Figure 5C,D). The data extracted from resistance measurements of the three different samples (numbered 1, 2, and 3) are plotted in the pressure–temperature phase diagram in Figure 3C. This is the first construction of the *P*-vs-*T* phase diagram of the TiS_3_ microstructure over such a large phase space and summarizes the unique sequence of electronic and structural transitions central to this work.

**Figure 4:**
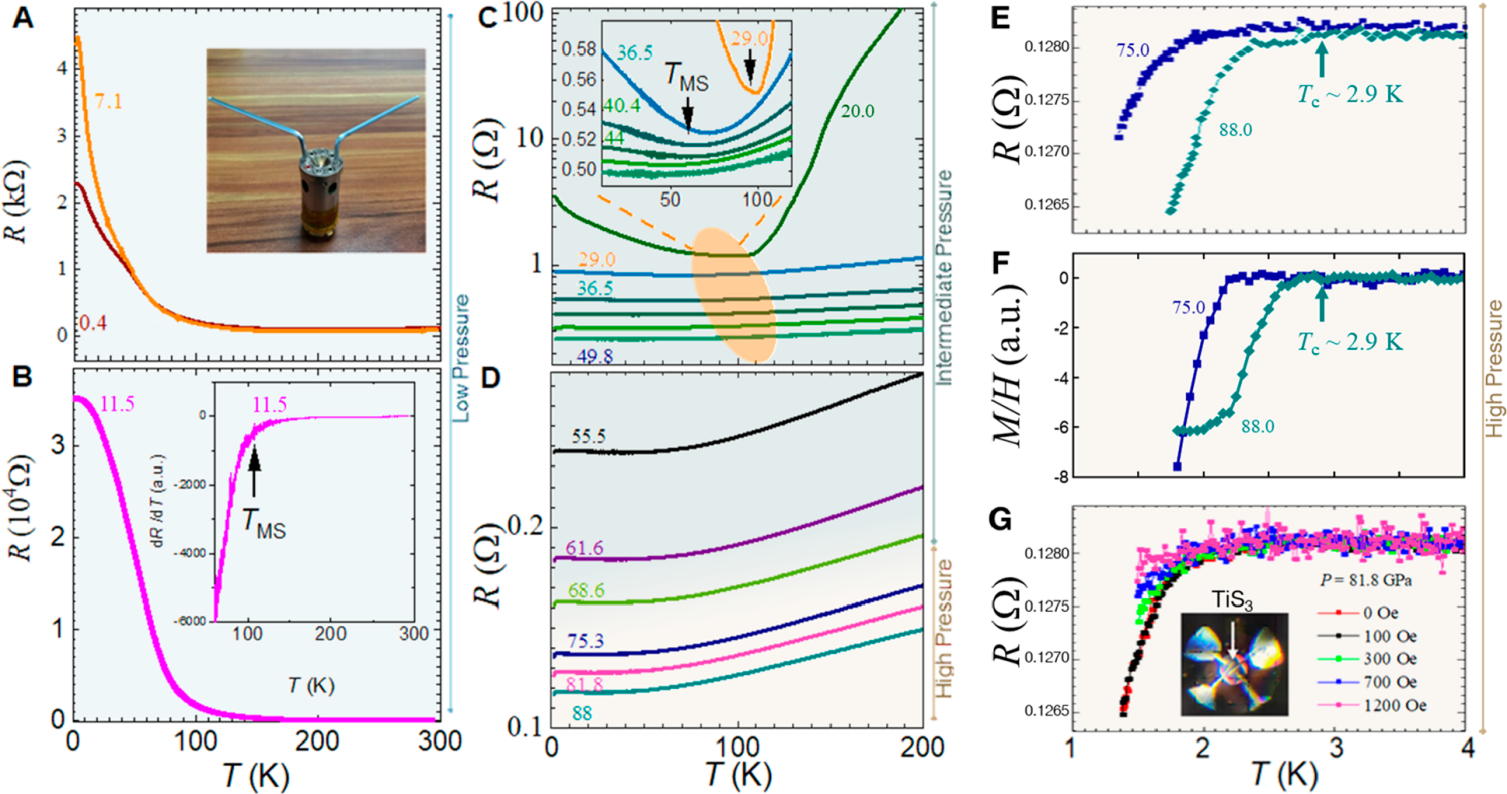
Temperature-dependent resistance of TiS_3_ (Sample 1) at different pressures. The pale blue, teal green, and beige plot backgrounds correspond to low-, intermediate-, and high-pressure regimes, respectively (as in Figure 3). Plots in (A, B, C, D, F, and G) are labeled using the applied pressures in units of GPa. (A, B) Resistance at pressures of 0.4, 7.1, and 11.5 GPa. Inset of (A) shows a pressure cell which was used for the high-pressure transport experiments. Inset of (B) illustrates the *T* derivative of *R* used in estimating the *T*_MS_. All resistance measurements in this work, including (A, C–F), use the *R*_||_ plan, i.e., the resistance is measured parallel to the *b*-axis. (C) Resistance under intermediate pressures, from 20.0 to 49.8 GPa. The inset shows the curves from the dashed purple rectangle, near *T*_MS_. Under increasing pressure, *T*_MS_ shifts to lower temperature. The 20 and 29 GPa curves are shifted vertically for clarity. (D) Resistance under intermediate and high pressures, from 55.5 to 88 GPa. (E) Zoomed-in curves from (D) at 75.3 and 88.0 GPa near the superconducting transitions. The 75.3 GPa curve is vertically shifted for clarity. (F) Temperature dependence of the DC magnetic susceptibility (*M*/*H*) measured in DC field with an amplitude of 30 Oe at elevated pressures of 75.0 and 88.0 GPa in arbitrary units (a.u.). (G) The *R*-vs-*T* curves at various magnetic fields under 81.8 GPa. The inset shows an image of the top view of TiS_3_ sample mounted in a diamond anvil cell.

**Figure 5:**
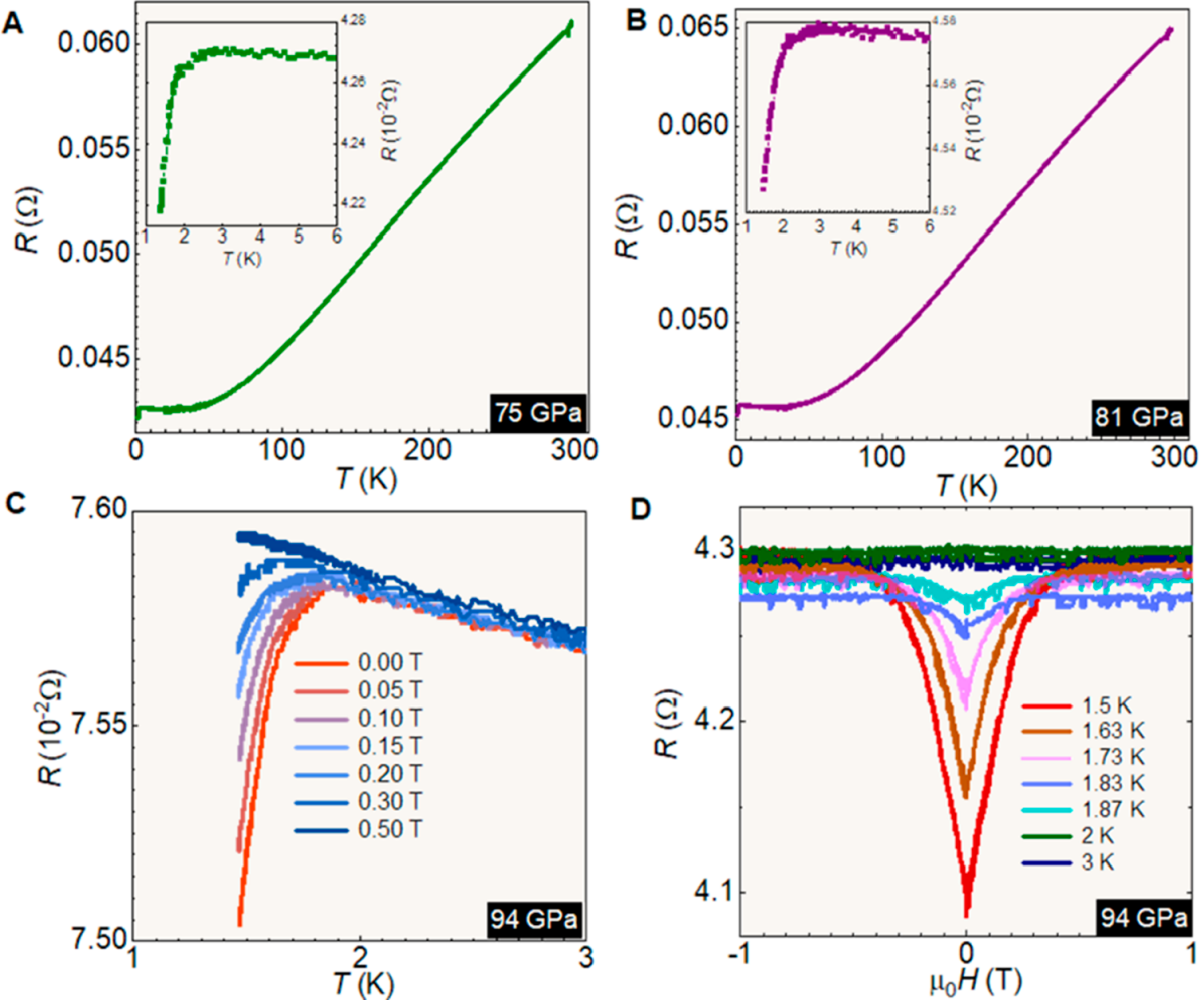
Temperature and field dependences of transport of TiS_3_ (Sample 2) in the high-pressure regime. Temperature dependence of the resistance between 300 and 1.5 K under (A) 75 GPa and (B) 81 GPa. The insets show the low-temperature parts of the curves. (C) Resistance as a function of temperature under different applied magnetic fields. (D) Resistance as a function of applied magnetic fields at different temperatures down to 1.5 K.

Detailed transport measurements for two samples are presented in Figures 4 and 5. The measurements for Sample 1 are shown in Figure 4, while those for Sample 2 are shown in Figure 5 and Figures S2 and S3 of the Supporting Information. Figure 4 shows the temperature dependence of the resistance for the TiS_3_ microstructure under various pressures up to 88 GPa for the whole temperature range of 1.5–200 K. For 0.0 ≤ *P* ≤ 11.5 GPa, the sample exhibited a semiconducting behavior (d*R*/d*T* < 0) at low *T* (Figure 4A,B). The resistance increased (Figure 3B) with *P* from ambient pressure to 7.1 GPa (Figure 4A) and continued to increase until 11.5 GPa (Figure 4B). This interesting behavior was atypical of a dielectric because one usually expects the application of pressure to decrease interatomic distances, thereby increasing the overlap of electronic orbitals and reducing its band gap and electrical resistance. As another example of such unusual behavior, pressure enhancement of insulating state had been also reported^41^ for Eu_2_Sn_2_O_7_, for which such a behavior had been attributed to the increase in trigonal lattice distortion. For TiS_3_ microstructure, this behavior was associated with the unique bonding of the S atoms, which under the application of pressure actually increased the S–S interatomic distance (i.e., unbuckling) (Figure 1A) of the low-pressure *P*2_1_/*m* (type-I) phase, as we will explain later.

At ∼11.5 GPa, the resistance experienced a sharp decrease (Figure 4C) upon further compression. At low temperatures (*T* < 100 K), this manifested as an insulator-to-semiconductor transition. Beyond 11.5 GPa, TiS_3_ exhibited metallic behavior (d*R/*d*T* > 0) above 100 K. Both behaviors persisted throughout the intermediate-pressure regime (11.5 to 73.2 GPa) (Figure 3c). For example, at 29.0 GPa, as shown in Figure 4C and the inset of it, a crossover to d*R/*d*T* > 0 was observed below 100 K, indicating the occurrence of metal-to-semiconductor transition. With increasing pressure, the metal-to-semiconductor transition temperature shifted to a lower temperature (Figure 3C,C inset), and the metallic behavior became dominant. Note that we define the metal-to-semiconductor transition (or crossover) temperatures, *T*_MS_, as the temperature at minimum of the measured resistance (see inset of Figure 4C). To rigorously define the exact point of a metal-to-semiconductor transition (if any), one should take into account the temperature dependence of electronic mobility.^18^ Summarizing our experiment and calculations for *P* < 30–39 GPa, we note that our results qualitatively confirm the conclusions of ref (4). Evidently, the state of TiS_3_ in this *P* range was very similar to the state at ambient pressure as described earlier, but at much lower energy scales. Hence, the *R*-vs-*T* dependence could similarly either be dominated by the interplay of carrier mobility and concentration or another state akin to a degenerate semiconductor. Thus, we cannot unambiguously establish if the metal-to-semiconductor transition is merely formal or true.

From Figure 3A and B, we see that changes in transport properties were accompanied by the isosymmetric structural transition from the *P*2_1_/*m* (type-I) phase to the *P*2_1_/*m* (type-II) phase at *P* ≈ 11.5 GPa. Evidently, as pressure increased from ambient pressure, the S–S distance (Figure 1A) increased and the band gap grew, explaining the atypical increase in resistance as pressure increased from ambient pressure to 7.1 GPa (Figure 4A). The compound became more and more insulating until the S–S bond broke and switched bond to the S atom from the neighboring chain (Figure 1B). The band gap then decreased abruptly, marking the onset of an insulating-to-semiconducting transition at low temperature (<∼100 K) (Figure 3C). Further increase in pressure caused the two S atoms to approach each other. Note that the switching of the S–S bond (from Figure 1A labeled in orange to Figure 1C labeled in purple) at 11.5 GPa did not change the number of bonds for each Ti atom, i.e., all four valence electrons of a Ti atom were still bonded to three S atoms, even though the valence and conducting bands became closer and potentially even overlapping as pressure increased. As a result, the valence electrons became more easily excited at *P* > 11.5 GPa. For a complete understanding of the energy structure evolution, one could in principle also track the change in donor level (or mini-band) under pressure, which is about 35 meV below the conducting band edge at ambient pressure.^1,6,42,43^

Figure 4E, F, and G provide evidence of a superconducting transition. When the pressure reached 73–75 GPa, the resistance of TiS_3_ started to drop as the temperature decreased below 2 K (Figure 4E). As pressure increased from 73 to 88 GPa, the drop in resistance became increasingly pronounced (Figure 4D,E), indicating a superconducting transition of *T*_c_ ≈ 2.9 K at 88 GPa. Figure 5A and B, respectively, show typical cool-down resistance curves for TiS_3_ under the high pressures of 75 and 81 GPa in the temperature range of 2–300 K. Upon cooling down from 300 K, the sample displayed a metallic behavior followed by superconducting transition at *T*_c_ of 1.9 and 2.9 K (defined at 90% of the normal-state resistance) with a width of transition of about Δ*T*_c_ = 0.1 and 0.15 K for *P* = 75 and 81 GPa, respectively. Since our samples did not contain any magnetic atoms, the observed drop in resistance could not be due to any magnetic states. In addition, the experimental transition (onset) temperature of 2.9 K agreed well with our calculated superconducting transition temperature, *T*_c_, which lay in the range of 2.5–5.5 K (see Supporting Information Sec. S1 for details). Since the resistance drop was only 12%, its association with superconductivity merits further investigation.

To further validate the emergence of pressure-induced superconductivity, we conducted magnetic experiments under high-pressure conditions. For these experiments, we employed a vibrating coil magnetometer in conjunction with a superconducting quantum interference device (SQUID) magnetometer to measure the DC magnetic susceptibility (see Supporting Information Sec. S4 for details). We measured the DC magnetic susceptibility as a function of temperature at two selected pressure points (Figure 4F). The emergence of a magnetic moment exhibiting negative magnetic susceptibility at low temperatures is a manifestation of the Meissner effect. The critical temperatures (*T*_c_), at which we recorded significant decreases in magnetic susceptibilities, were consistent with those of the resistance measurement (Figure 4E), underscoring the robustness of our results. We further measured the temperature-dependent resistance under various external out-of-plane magnetic fields, *H*, at 81.8 GPa (Figure 4G). As expected, the superconducting transition shifted continuously toward lower temperatures with increasing magnetic field. In Figure 5C and D, we report the *R*-vs-*H* (0–0.5 T) and *R*-vs-*T* (1.5–3.0 K) measurements at *P* = 94 GPa. The validity of our findings is bolstered by their reproducibility across different TiS_3_ samples: sharp and conspicuous drops in resistance were recorded for two samples at similar high-pressure low-temperature conditions. This intriguing correlation became even more compelling when the samples were subjected to very high magnetic fields, which effectively obliterated any manifestations of the superconducting transition. High *H* values returned the *R*(*T*) behavior to that of the dielectric type that was observed at higher temperatures (Figures 4G and 5C) and the emergence of magnetoresistance involved the suppression of the low-temperature anomaly. If the anomaly were of a nonsuperconducting nature, *R*(*T*) would only be modified by high *H*. We conclude that under high pressure, superconductivity at low temperature is a general feature of TiS_3_ of, at least, incipient and probably filamentary^44^ type.

## Supporting Information Available

Summary of this work, computational details of first-principles calculations, additional experimental details related to growth and synthesis of TiS_3_ microstructures, descriptions of Matthias rules, additional experimental details related to transport measurements under high pressure (PDF)
