# Supplementary material for: From Insulator to Superconductor: A Series of Pressure-Driven Transitions in Quasi-One-Dimensional TiS3 Nanoribbons
Source: Nano Lett. 2024 Apr 29;24(18):5562–9. doi: 10.1021/acs.nanolett.4c00824 (PMC11082921; doi:10.1021/acs.nanolett.4c00824)
Supplement: Supplementary file 1 — nl4c00824_si_001.pdf [file nl4c00824_si_001.pdf]

Supporting Information for

From insulator to superconductor: a series of pressure-driven transitions in a quasi-one-dimensional TiS_3 nanoribbons

Mahmoud Abdel-Hafiez[§,€,±,*], Li Fen Shi[$,#], Jinguang Cheng[$,#], Irina G. Gorlova[⊥], Sergey G. Zybtsev[⊥], Vadim Ya. Pokrovskii[⊥], Lingyi Ao[‡], Junwei Huang[‡], Hongtao Yuan[‡], Alexsandr N. Titov[∇], Olle Eriksson[±], and Chin Shen Ong[±,*]

[§] Center for Advanced Materials Research, Research Institute of Sciences and Engineering, University of Sharjah

[€] Department of Applied Physics and Astronomy, University of Sharjah, P. O. Box 27272 Sharjah, United Arab Emirates

[$] Beijing National Laboratory for Condensed Matter Physics and Institute of Physics, Chinese Academy of Sciences, Beijing 100190, China

[#] School of Physical Sciences, University of Chinese Academy of Sciences, Beijing 100190, China

[⊥] Kotelnikov Institute of Radioengineering and Electronics of RAS, 125009 Moscow, Russia

[‡]National Laboratory of Solid State Microstructures, College of Engineering and Applied Sciences and Jiangsu Key Laboratory of Artificial Functional Materials, Nanjing University, Nanjing 210000, China.

[∇] M.N. Miheev Institute of Metal Physics of Ural Branch of Russian Academy of Sciences, 620990 Yekaterinburg, Russia

*Corresponding authors. Emails: mahmoud.hafiez@physics.uu.se, chinshen.ong@physics.uu.se

Contents

Summary

S1. First-Principles Calculations

S2. Growth and Synthesis of TiS_3 Microstructures

S3. Matthias rules

S4. Transport Measurements under High Pressure

Summary

Our results demonstrate the non-trivial evolution of TiS3 microstructures under pressure: transitioning from an insulating to a superconducting state, through various electronic and lattice orders. Firstly, by increasing from ambient pressure up to ~12 GPa, the insulating state became enhanced with pressure, consistent with Ref. [1]. Subsequently, an isosymmetric isoelectronic transition to a semiconducting state occurred around 20 GPa. With further increase in pressure, TiS3 switched into a cubic crystal structure at around 70 GPa, becoming metallic. We present experimental evidence supporting the superconducting behavior of this phase below 2–3 K, corroborated by first-principles theoretical calculations.

S1. First-Principles Calculations

The density functional theory (DFT) and density functional perturbation theory (DFPT) calculations were carried out using the Quantum ESPRESSO [2] package, which uses a plane-wave basis set. The plane-wave cut-off for the DFT calculation was set at 35 Ry for the plane-wave

expansion of the wave functions. For the monoclinic $P2_1/m$ (type-I), monoclinic $P2_1/m$ (type-II) and cubic $Pm\overline{3}n$ crystal phases, **k**-grids of 7×10×4, 8×10×4 and 10×10×10 were, respectively, used for the self-consistent DFT calculations. Gaussian smearing was used when integrating over the Brillouin zone. Our calculation uses scalar-relativistic GBRV ultra soft pseudopotentials [3,4] with nonlinear core correction [5]. The generalized gradient approximation (GGA-PBE) was used for the DFT exchange-correlation functional. In Fig. 3A of the Main Text, the crystal structures at each pressure point were obtained by allowing the atomic positions and the crystal shapes to relax for a particular fixed crystal volume. Atomic relaxations were performed until all components of all forces were minimized within the convergence threshold of 1.0E-5 Ry/bohr and the total energy is also minimized within the convergence threshold of 1.0E-8 Ry. The cell shape relaxation was performed until the pressure of the cell is converged within the threshold of 5E-5 GPa. For the $P2_1/m$ (type-I), the relaxed crystal structure at ambient pressure (i.e., 0.0 GPa) is calculated to have the lattice parameters of a = 5.030 Å, b = 3.417 Å, c = 9.640 Å, α = γ = 90.00° and β = 95.94°. The calculated band structure is semiconducting, as shown in Fig. S1. For the $P2_1/m$ (type-II), the relaxed crystal structure at 14.6 GPa (in the intermediate pressure regime) is calculated to have the lattice parameters of a = 4.527 Å, b = 3.267 Å, c = 8.157 Å, α = γ = 90.00° and β = 97.68°. For Figs. S1B-D, the high-pressure $Pm\overline{3}n$ cubic crystal phase with a lattice parameter of 4.391 Å and relaxed atomic positions was used. The calculated pressure using this lattice parameter is 78.8 GPa.

The phonons are calculated on a regular **q**-grid of 4×4×4 using DFPT and Fourier-interpolated to obtain the phonon band structure in Fig. S1D. When calculating the electron-phonon mass enhancement parameter, λ and solving the McMillan-Allen-Dynes equation[6,7], the optimized tetrahedron method[8] is used to perform integrations over the Brillouin zone, and an

interpolated **k**-grid of 16×16×16 was used for the wavefunctions. The **q**-grid was also shifted by half a grid step in each direction when λ was calculated. By calculating the phonon modes (Fig. 1H) using density function perturbation theory (DFPT) and solving the McMillan-Allen-Dynes equation[6,7] using a retarded Coulomb repulsion μ^* between 0.10 and 0.15, the phonon-mediated superconducting transition temperature, T_c, is calculated to lie between 5.5 K and 2.5 K, respectively. This is in close agreement with the T_c of 2.9 K measured in experiment, as described in the Main Text.

S2. Growth and Synthesis of TiS_3 Microstructures

The TiS_3microstructures were grown by chemical vapor-transport techniques[9]. All preparation and storage steps were carried out in an Ar-filled glovebox, the O_2 and H_2O level is less than 0.1 ppm. Quartz ampoules by gas transport reactions using TiS_2 as the initial batch and sulfur excess are used. Transfer was performed to the cold end on which a temperature of 500°C was maintained whereas the TiS_2 weighed portion was kept at a temperature of 700 °C. The samples were faceted microstructures with the dimensions b = 500-3000μm, a = 10-200μm and c = 1-20 μm. The good quality of the microstructures is confirmed from x-ray diffraction[10] and high resolution scanning transmission electron microscopy[11].

S3. Matthias rules

The Matthias' rules[12] are a set of empirical guiding principles predicting superconductivity in new materials. Unlike the intermediate-pressure $P2_1/m$ (type-II) phase, the high-pressure cubic phase satisfies all of the Matthias rules.

(1) The high-pressure phase has a cubic crystal structure. The high degree of symmetry of the cubic lattice leads to the existence of pronounced nesting vectors between the S 3*p* bands forming the Fermi surface in the first Brillouin Zone, as shown in Fig. S1C.

(2) It is a metal with *d*-electrons in the valence band. In Fig. 1F, we plot the band structure of TiS_3 on a color scale corresponding to the projections of the wavefunctions onto the atomic pseudo wavefunctions of the S 3*p* (blue) and Ti 3*d* orbitals (red). It is clear from the figure that some of the bands at the Fermi level around the M point have strong 3*d* characters.

(3) The high-pressure phase is not in the vicinity of ordered magnetism.

(4) The high-pressure phase is not near a metal-to-insulator transition, which takes place only at the pressure of ~11.5 GPa.

We note that despite the historical importance of the Matthias' rules, they were proposed even before the Bardeen-Cooper-Schrieffer (BCS) theory[13] was published. Hence, while they have been remarkably successful in predicting and explaining a wide range of experimental observations related to superconductivity, they are unable to fully account for some superconducting phenomena such as high-temperature and unconventional superconductivity.

S4. Transport and Magnetic Measurements under High Pressure

The high-pressure resistance of TiS_3microstructure was measured by standard four-probe method in a non-magnetic Cu-Be diamond anvil cell of 200 μm culet. A rhenium gasket was pre-indented to ~33 μm, and then a 55 μm-diameter hole was drilled in the center using a laser-drilling system. The rhenium gasket was covered with a c-BN epoxy insulating layer. A piece of the TiS_3 microstructure was placed at the center of the sample chamber filled with KBr pressure

transmitting medium. The pressure was determined by R1 fluorescence line of ruby in the lower-pressure range, and the Raman spectrum of diamond was employed as a pressure calibrant in the higher-pressure region.

Figure S2C represents pressure-dependent resistance values at temperature being 2 K, 100 K, 150 K, and 300 K for sample No 2 According to the different behaviors of resistance values at different temperatures at the same pressure, the pressure range of 0 GPa to 100 GPa could be divided into three areas. The three areas might be associated with three distinct crystal structures.

Figure S4 shows the temperature dependence of resistance for another TiS_3 sample (Sample 3) under various pressures up to 98.0 GPa within the whole temperature range of 1.5-300 K. In Fig. S2A, we see that for $0.4 \leq P \leq 11.8$ GPa, the sample exhibits a semiconducting behavior ($dR/dT < 0$), and the resistance is enhanced substantially at 10.4 GPa. Upon further compression, the resistance begins to decrease and exhibits a metallic behavior at high temperature.

At 20.0 GPa, as shown in Fig. S4B, upon cooling a partial upturn of resistance ($dR/dT > 0$) is observed below 110 K, indicating the occurrence of metal-to-semiconductor transition (like at $P = 0$). The metallic behavior becomes dominant, and the metal-to-semiconductor transition temperature moves to lower temperatures as the pressure increases. Interestingly, a superconducting transition with the onset temperature $T_c \sim 2$ K is observed at 55.5 GPa (see Fig. S2D). The onset of superconductivity reaches of ~3 K at 61.6-68.6 GPa and then begins to decrease with pressure. To confirm the occurrence of superconductivity, we have also measured the temperature-dependent resistance under various external magnetic fields at 85.0-98.0 GPa (Figs. 3A-D of Main Text) for this sample (Sample 2). As expected, the superconducting transition continuously shifts to the lower temperatures with increasing magnetic field.

To further validate the emergence of pressure-induced superconductivity within our studied system, we conducted magnetic experiments under high-pressure conditions. For these experiments, we employed a vibrating coil magnetometer in conjunction with a superconducting quantum interference device (SQUID) magnetometer to measure the DC magnetic susceptibility. Pressure levels of approximately 98 GPa, estimated at room temperature, were achieved using a specialized miniature diamond anvil cell (mDAC) composed of CuBe. This mDAC was designed to be compatible with the SQUID magnetometer, facilitating seamless integration[14-15].

References and Notes

(1) An, C.; Lu, P.; Chen, X.; Zhou, Y.; Wu, J.; Zhou, Y.; Park, C.; Gu, C.; Zhang, B.; Yuan, Y.; Sun, J.; Yang, Z. Pressure-Induced Anomalous Enhancement of Insulating State and Isosymmetric Structural Transition in Quasi-One-Dimensional Ti S3. *Phys. Rev. B* **2017**, *96* (13), 134110. https://doi.org/10.1103/PhysRevB.96.134110.

(2) Giannozzi, P.; Baroni, S.; Bonini, N.; Calandra, M.; Car, R.; Cavazzoni, C.; Ceresoli, D.; Chiarotti, G. L.; Cococcioni, m.; Dabo, I.; Corso, A. D.; Fabris, S.; Fratesi, G.; de Gironcoli, S.; Gebauer, R.; Gerstmann, U.; Gougoussis, C.; Kokalj, A.; Lazzeri, M.; Martin-Samos, L.; Marzari, N.; Mauri, F.; Mazzarello, R.; Paolini, S.; Pasquarello, A.; Paulatto, L.; Sbraccia, C.; Scandolo, S.; Sclauzero, G.; Seitsonen, A. P.; Smogunov, A.; Umari, P.; Wentzcovitch, R. M. Quantum ESPRESSO: A Modular and Open-Source Software Project for Quantum Simulations of Materials. *J. Phys. Condens. Matter* **2009**, *21*, 395502. https://doi.org/10.1088/0953-8984/21/39/395502.

(3) Vanderbilt, D. Soft Self-Consistent Pseudopotentials in a Generalized Eigenvalue

Formalism. *Phys. Rev. B* **1990**, *41* (11), 7892–7895. https://doi.org/10.1103/PhysRevB.41.7892.

(4) Garrity, K. F.; Bennett, J. W.; Rabe, K. M.; Vanderbilt, D. Pseudopotentials for High-Throughput DFT Calculations. *Comput. Mater. Sci.* **2014**, *81*, 446–452. https://doi.org/10.1016/j.commatsci.2013.08.053.

(5) Louie, S. G.; Froyen, S.; Cohen, L.; Cohen, M. L. Nonlinear Ionic Pseudopotentials in Spin-Density-Functional Calculations. *Phys. Rev. B* **1982**, *26* (4), 1738–1742. https://doi.org/10.1103/PhysRevB.26.1738.

(6) McMillan, W. L.; Rowell, J. M. Lead Phonon Spectrum Calculated from Superconducting Density of States. *Phys. Rev. Lett.* **1965**, *14* (4), 108. https://doi.org/10.1103/PhysRevLett.14.108.

(7) Allen, P. B.; Dynes, R. C. Transition Temperature of Strong-Coupled Superconductors Reanalyzed. *Phys. Rev. B* **1975**, *12* (3), 905–922. https://doi.org/10.1103/PhysRevB.12.905.

(8) Kawamura, M.; Gohda, Y.; Tsuneyuki, S. Improved Tetrahedron Method for the Brillouin-Zone Integration Applicable to Response Functions. *Phys. Rev. B - Condens. Matter Mater. Phys.* **2014**, *89* (9), 1–8. https://doi.org/10.1103/PhysRevB.89.094515.

(9) Shkvarin, A. S.; Yarmoshenko, Y. M.; Yablonskikh, M. V.; Merentsov, A. I.; Titov, A. N. An X-Ray Spectrscopy Study of the Electronic Structure of TiS3. *J. Struct. Chem. 2014 556* **2015**, *55* (6), 1039–1043. https://doi.org/10.1134/S0022476614060067.

(10) Bolotina, N. B.; Gorlova, I. G.; Verin, I. A.; Titov, A. N.; Arakcheeva, A. V. Defect Structure of TiS3 Single Crystals of the A-ZrSe3 Type. *Crystallogr. Reports 2016 616*

2016, *61* (6), 923–930. https://doi.org/10.1134/S1063774516060055.

(11) Trunkin, I. N.; Gorlova, I. G.; Bolotina, N. B.; Bondarenko, V. I.; Chesnokov, Y. M.; Vasiliev, A. L. Defect Structure of TiS3 Single Crystals with Different Resistivity. *J. Mater. Sci.* **2021**, *56* (3), 2150–2162. https://doi.org/10.1007/S10853-020-05357-0/TABLES/4.

(12) Matthias, B. T. Empirical Relation between Superconductivity and the Number of Valence Electrons per Atom. *Phys. Rev.* **1955**, *97* (1), 74–76. https://doi.org/10.1103/PhysRev.97.74.

(13) Bardeen, J.; Cooper, L. N.; Schrieffer, J. R. Microscopic Theory of Superconductivity. *Phys. Rev.* **1957**, *106* (1), 162–164. https://doi.org/10.1103/PhysRev.106.162.

(14) M. Mito, M. Hitaka, T. Kawae, K. Takeda, T. Kitai, andN. Toyoshima, Jpn. J. Appl. Phys. 40, 6641 (2001).

(15) Y. Kvashnin, D. VanGennep, M. Mito, S. A. Medvedev, R. Thiyagarajan, O. Karis, A. N. Vasiliev, O. Eriksson, and M. Abdel-Hafiez, *Coexistence of Superconductivity and Charge Density Waves in Tantalum Disulfide: Experiment and Theory*. Phys. Rev. Lett. 125, 186401 – Published 27 October 2020

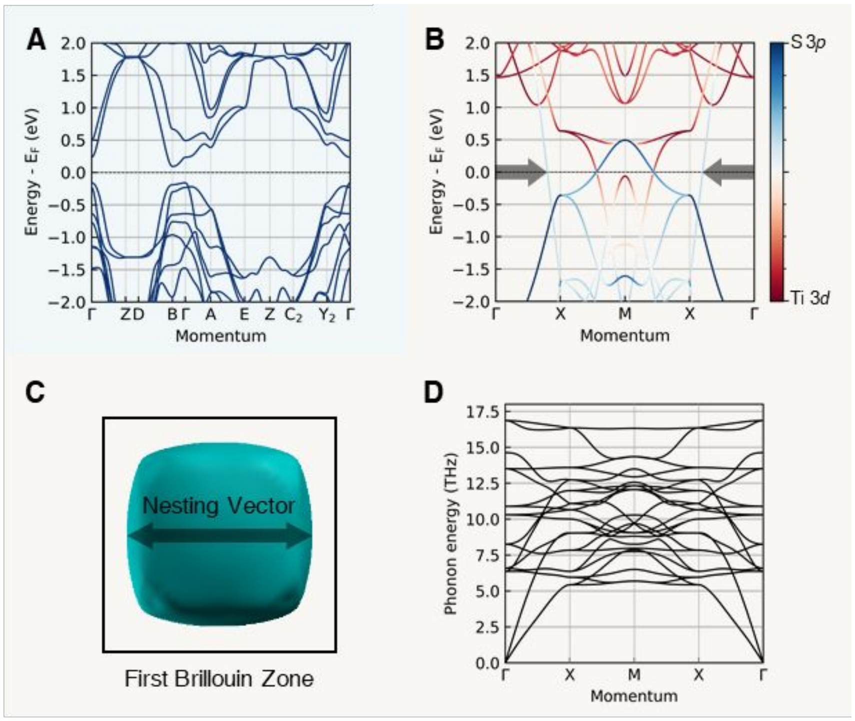

Fig. S1. DFT and DFPT Calculations. (**A**) Calculated DFT bandstructurefor the $\boldsymbol{P2_1/m}$ (type-I) low-pressure phase at 2.0 GPa.(**B**) DFT band structure of the high-pressure phase at 78.8 GPa. The band states are projected onto the atomic pseudo wavefunction of S 3*p* and Ti 3*d* orbitals, with the color bar representing the degree of projection. E_F stands for the Fermi energy. The arrow shows the nesting vector annotated in (C), connecting two S states on the Fermi surface. (**C**) Nested part of the Fermi surface, originating from the S 3*p* bands in the first Brillouin Zone as shown in (B). (**D**) The calculated phonon band structure of the cubic phase at 78.8 GPa.

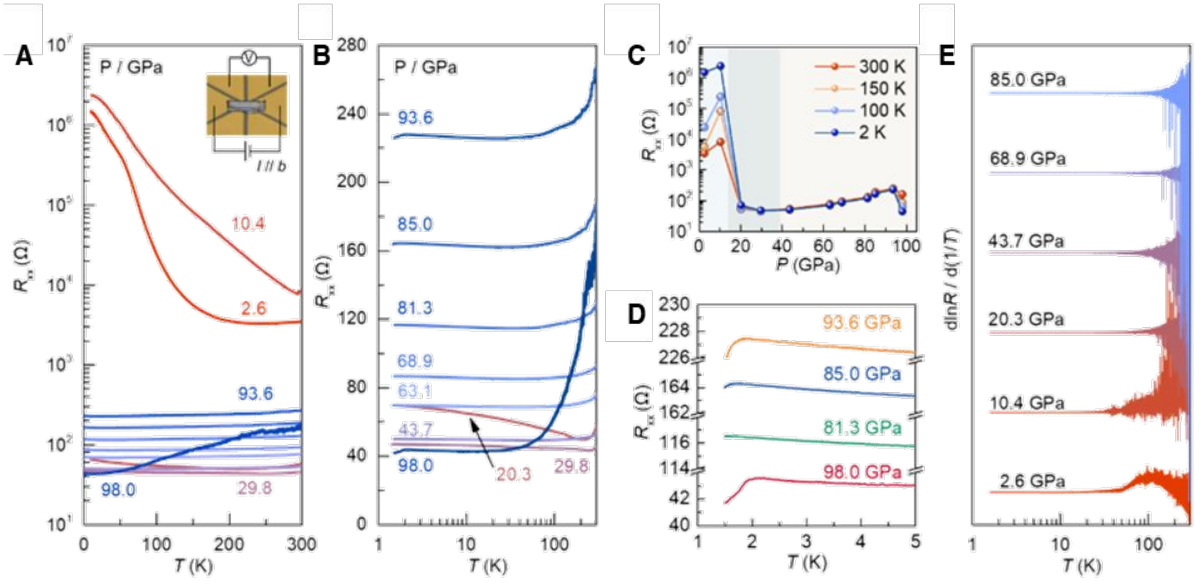

Fig. S2. Resistance of TiS_3 (Sample 2) microstructures under various pressures and temperatures (**A**) $\boldsymbol{R_{xx}}$-$\boldsymbol{T}$ relation from 1.6 K to 300 K under the pressure ranging from 2.6 GPa to 98.0 GPa. Inset shows the optical image of the device, and the direction of electrical current is parallel to *b*-axis of the crystal structure. (**B**) $\boldsymbol{R_{xx}}$-$\boldsymbol{T}$ relation from 1.6 K to 300 K under the pressure ranging from 20.3 GPa to 98.0 GPa. (**C**) Pressure-dependent $\boldsymbol{R_{xx}}$ values with temperature being 2 K, 100 K, 150 K, and 300 K, respectively. (**D**) Zoom-in of $\boldsymbol{R_{xx}}$-$\boldsymbol{T}$ relation from 1.6 K to 5 K with the pressure ranging from 81.3 GPa to 98.0 GPa. Note that the resistance drops at $\boldsymbol{T \sim 1.7}$ **K**starts to be observable at 85.0 GPa, and that such a feature becomes increasingly apparent as the pressure increases. (**E**) Temperature dependence of d ln *R*/d(1/*T*) under different pressures.

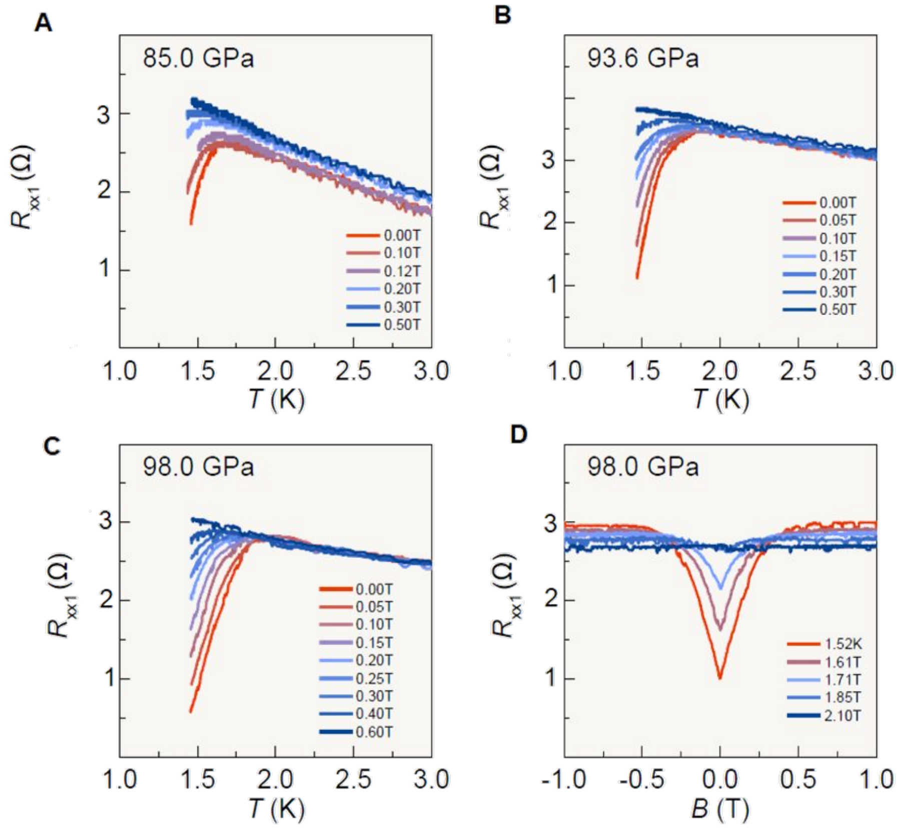

Fig. S3. Temperature dependent resistance of TiS_3 (Sample 2) microstructures under high pressure. (**A**) 85 GPa, (**B**) 93 GPa, and (**C**) 98 GPa under various temperature. (**D**) For the pressure of 98 GPa, the resistance has also been measured as a function of applied fields at different temperatures.

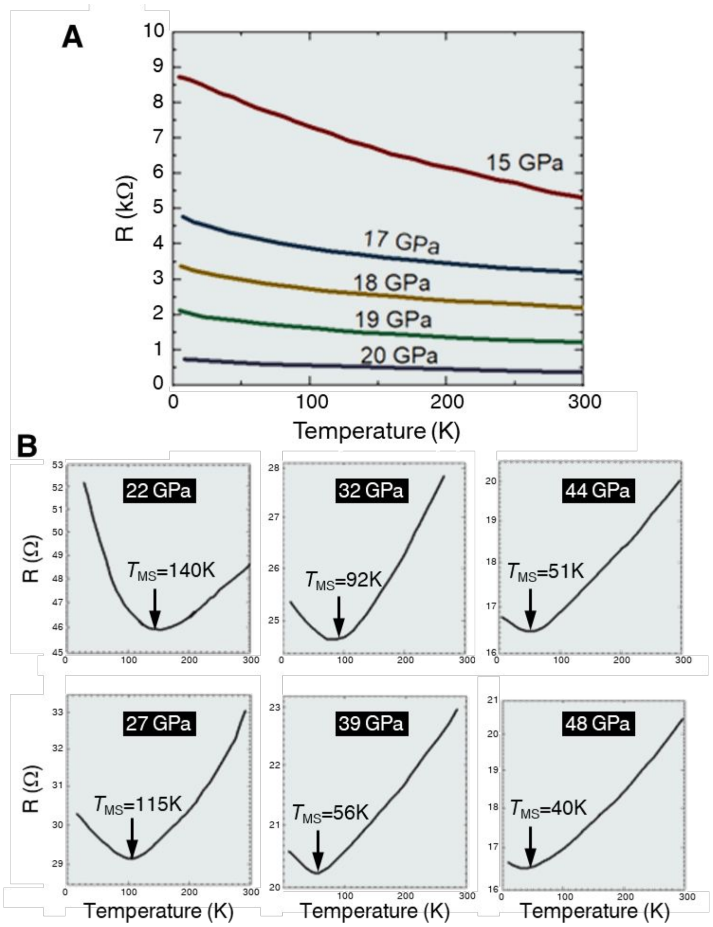

Fig. S4. Temperature dependent resistance of TiS_3 (Sample 3) microstructures under various pressures. (**A**) Pressure ranging from 15 GPa to 20 GPa. (**B**) Pressure ranging from 22 GPa to 48 GPa.

Peaks	Recorded Polarization	Wave number (cm^{-1})	dω/dT ($cm^{-1}.K^{-1}$)	Total anharmonicity (10^{-5} K^{-1})
I: A_g^{rigid}	Case (i)	174	-0.01	-5.75
	Case (ii)	174	-0.008	-4.59
II: $A_g^{internal}$	Case (i)	298	-0.02	-6.71
	Case (ii)	299	-0.01	-3.34
III: $A_g^{internal}$	Case (i)	367	-0.02	-5.44
	Case (ii)	370	-0.016	-4.35
IV: A_g^{S-S}	Case (i)	556	-0.02	-3.59
	Case (ii)	557	-0.012	-2.15

Table S1. Shifting rates of the four main Raman modes with temperature and total anharmonicities of respective Raman modes in both cases of E ⊥ D and E || D.
